# Haloperidol for Hypoactive and Hyperactive Delirium in ICU Patients—A Post Hoc Bayesian Analysis of the AID‐ICU Trial

**DOI:** 10.1111/aas.70336

**Published:** 2026-09-18

**Authors:** Nina C. Andersen‐Ranberg, Lone Musaeus Poulsen, Anders Perner, Johanna Hästbacka, Matthew Morgan, Giuseppe Citerio, Stine Estrup Damby, Camilla Bekker Mortensen, Marie Oxenbøll Collet, Sven‐Olaf Weber, Anne Sofie Andreasen, Morten Bestle, Bülent Uslu, Helle Scharling Pedersen, Louise Gramstrup Nielsen, Kjeld Damgaard, Troels Bek Jensen, Trine Sommer, Nilanjan Dey, Ole Mathiesen, Anders Granholm

**Affiliations:** ^1^ Department of Anaesthesiology and Intensive Care Medicine Zealand University Hospital Køge Denmark; ^2^ Collaboration for Research in Intensive Care (CRIC) Copenhagen Denmark; ^3^ Department of Intensive Care Copenhagen University Hospital – Rigshospitalet Copenhagen Denmark; ^4^ Department of Clinical Medicine Copenhagen University Copenhagen Denmark; ^5^ Department of Anesthesia and Intensive Care, Tampere University Hospital Wellbeing Services County of Pirkanmaa, Faculty of Medicine and Health Technology, Tampere University Tampere Finland; ^6^ Department of Intensive Care Cardiff University Hospital of Wales Cardiff UK; ^7^ Neuro‐Intensive Care, Department of Neurosciences San Gerardo Hospital, Fondazione IRCCS San Gerardo dei Tintori Monza Italy; ^8^ School of Medicine and Surgery University of Milan‐Bicocca Milan Italy; ^9^ Department of Anaesthesia and Intensive Care Aalborg University Hospital Aalborg Denmark; ^10^ Department of Anaesthesiology and Intensive Care Copenhagen University Hospital – Herlev Herlev Denmark; ^11^ Department of Anaesthesiology and Intensive Care Copenhagen University Hospital – North Zealand Hillerød Denmark; ^12^ Department of Anaesthesiology and Intensive Care Medicine Zealand University Hospital Roskilde Denmark; ^13^ Department of Anaesthesiology and Intensive Care Nykøbing Falster Sygehus Nykøbing Falster Denmark; ^14^ Department of Anaesthesiology and Intensive Care Odense University Hospital Odense Denmark; ^15^ Department of Anaesthesiology and Intensive Care Hjørring Hospital Hjørring Denmark; ^16^ Department of Anaesthesiology and Intensive Care Gødstrup Hospital Herning Denmark; ^17^ Department of Anaesthesiology Hospital of Southern Jutland Aabenraa Denmark; ^18^ Section of Biostatistics, Department of Public Health University of Copenhagen Copenhagen Denmark

## Abstract

**Background:**

Delirium is clinically classified into hypoactive or hyperactive motoric subtypes, which may have different responses to treatment. We assessed heterogeneity in treatment effects according to baseline motoric subtype in the AID‐ICU trial, which randomised adult ICU patients with delirium to haloperidol versus placebo.

**Methods:**

In this post hoc study of the AID‐ICU trial, we used adjusted Bayesian linear and logistic regression models to assess potential differential effects of haloperidol on 90‐day outcomes according to motor subtypes. Effect estimates are reported as absolute differences within and between subtypes (mean differences (MD)/risk differences (RD) and differences in MDs/RDs), with probabilities of any benefit, any harm and differential effects between subtypes.

**Results:**

Of 987 participants, 540 (54.7%) had hypoactive and 447 (45.3%) had hyperactive delirium at randomisation. Haloperidol was associated with more days alive out of hospital in both subgroups, with a 76.5% probability of a larger intervention effect in those with hyperactive delirium (difference in MDs 2.9 days; 95% CrI −4.8 to 10.6). Similar results were obtained for days alive without delirium/coma and days alive without mechanical ventilation. Haloperidol was associated with lower mortality in both subgroups, with a 73% probability of larger benefit in hyperactive delirium (difference in RDs −3.7%‐points; 95% CrI −15.3 to 7.8). We found no relevant differences in serious adverse reactions or use of rescue medications.

**Conclusion:**

Compared with placebo, haloperidol was associated with moderate to high probabilities of benefit in both hypoactive and hyperactive delirium, with moderate probabilities of greater effects in hyperactive delirium. Further research is warranted to confirm these results.

**Editorial Comment:**

In this post hoc analysis of the AID‐ICU trial, haloperidol was associated with moderate to high probabilities of benefit in both hypoactive and hyperactive delirium, with a tendency towards greater treatment effects in hyperactive delirium.

**Trial Registration:** ClinicalTrials.gov number: NCT03392376; EudraCT number, 2017‐003829‐15

## 1 Introduction

Delirium is common amongst critically ill patients in the intensive care unit (ICU) and is associated with increased morbidity and mortality [1, 2, 3, 4]. The clinical manifestation of delirium varies by motor subtype, typically classified as hypoactive and hyperactive delirium [5]. Hypoactive delirium is characterised by reduced responsiveness, lethargy and withdrawal, whereas hyperactive delirium presents with restlessness, agitation and sometimes aggression [5]. Patients may fluctuate between these states, termed mixed delirium [6]. Despite these differences, all delirium motor subtypes share core symptoms such as confusion and inattention [7].

The distinct clinical phenotypes of delirium motor subtypes suggest differences in underlying neurobiology [8]. Hyperactive delirium has been associated with increased dopaminergic activity and relative cholinergic deficiency, whereas hypoactive delirium may be more closely related to reduced arousal and global cerebral dysfunction. These differences raise the possibility that pharmacological treatments targeting dopaminergic pathways, such as antipsychotics, may have differential effects across delirium subtypes. However, the effects of commonly used antipsychotics on these subtypes are less studied [9]. A recent clinical practice guideline highlighted the need to evaluate potential heterogeneity in treatment effects [10].

Haloperidol, the most frequently used agent to treat delirium in ICU patients, was recently evaluated in the Agents Intervening against Delirium in the Intensive Care Unit (AID‐ICU) trial [11]. This randomised clinical trial compared haloperidol with placebo for delirium treatment in adult ICU patients. Whilst the primary outcome did not reach statistical significance (mean difference [MD] 2.9 days; 95% credible interval [CrI] −1.2 to 7.0; *p* = 0.22), the trial results were more compatible with beneficial effects of haloperidol than with harm, notably with lower mortality in the haloperidol group (risk difference [RD] −6.9%‐points; 95% CrI −13.0 to −0.6; *p* = 0.03), and the secondary Bayesian analysis suggested relatively high probabilities (> 92%) of benefit across multiple outcomes [11, 12]. The trial included 540 patients with hypoactive and 447 patients with hyperactive delirium at randomisation, providing an opportunity to explore potential heterogeneity in treatment effects.

In this post hoc analysis of the AID‐ICU trial, we aimed to assess whether the effects of haloperidol versus placebo differ according to delirium motor subtype at randomisation. We hypothesised that haloperidol would be associated with greater benefit in patients with hyperactive delirium compared with patients with hypoactive delirium.

## 2 Methods

This is a post hoc study of the AID‐ICU trial and results should therefore be considered as exploratory. The protocol and statistical analysis plan were publicly registered before conducting the analyses [13]. This manuscript adheres to the Strengthening the Reporting of Observational studies in Epidemiology (STROBE) [14] statement (completed checklist in Supporting Information S1: STROBE checklist) and the analyses are reported according to the Reporting Of Bayes Used in clinical STudies (ROBUST) guideline (with the exception that sensitivity analyses for the chosen priors were not conducted, as this was not considered relevant in the context of this post hoc study due to the use of weakly informative priors) [15].

### 2.1 The AID‐ICU Trial

The AID‐ICU trial was a multicentre, blinded, placebo‐controlled, centrally randomised clinical trial, stratified by site and delirium motor subtype [16]. The trial was approved by the competent authorities in all participating countries and included 1000 acutely admitted ICU patients with delirium at 18 sites in Denmark, Finland, the United Kingdom and Italy between 14 June 2018 and 9 April 2022. Eligible patients were randomised to receive intravenous haloperidol 2.5 mg three times daily and as‐needed doses up to a maximum of 20 mg daily or matching placebo (isotonic saline). Participants received trial medication as long as they were delirious. Patients were screened twice daily for delirium using either the Confusion Assessment Method of the Intensive Care Unit (CAM‐ICU) [17] or the Intensive Care Delirium Screening Checklist (ICDSC) [18]. The intervention period was from randomisation until discharge, death or a maximum of 90 days. Further details are available in the primary trial publication [11] and protocol [16, 19].

### 2.2 Population and Outcomes

This post hoc study assessed 90‐day outcomes in the intention‐to‐treat population of the AID‐ICU trial (*n* = 987), excluding participants before unblinding, that did not receive the intervention [20]. No formal sample size calculation was performed for this post hoc analysis. We assessed the following outcomes, all 90 days after randomisation:

– Days alive and out of the hospital.
– Mortality.
– Days alive without delirium or coma in the ICU.
– Days alive without mechanical ventilation.
– Number of patients with one or more serious adverse reactions (SARs) in the ICU.
– Number of patients using rescue medications (use of benzodiazepines, propofol or alpha‐2 agonists to treat delirium).
– Days with rescue medications.

Death within the 90‐day intervention period was not penalised to zero in the count outcomes [21]. The numbers reflect the actual days alive irrespective of survival status later in the 90‐day follow‐up period.

### 2.3 Statistical Analyses

All analyses were conducted using the R statistical software, version 4.4.1 (R Core Team, R Foundation for Statistical Computing, Vienna, Austria) using the *Tidyverse* packages and Stan through the *brms* package [22, 23, 24].

Baseline and outcome variables are presented stratified by treatment group (haloperidol or placebo) and delirium motor subtype (hypoactive or hyperactive) at randomisation. Descriptive data are presented as medians with interquartile ranges (IQRs) for numerical variables and presented as counts with percentages for categorical variables.

We used Bayesian linear regression models for all count outcomes and Bayesian logistic regression models for all dichotomous outcomes. The analyses were adjusted for stratification variables (site and delirium motor subtype) and included an interaction term between intervention and delirium motor subtype. Weakly informative priors encompassing all plausible effect sizes (detailed in Supporting Information S1: Calculation of effect estimates) were used. These priors have minimal influence on the results, as the posteriors are dominated by the data.

Results are reported as absolute differences, that is, MDs for numerical outcomes and RDs for binary outcomes. All effect estimates are presented as sample‐average (marginal) treatment effects, which were calculated using the joint posterior distributions from the models in a G‐computation procedure [25]. Specifically, we estimated predicted outcomes for each delirium motor subgroup under the assumption that all participants in the subgroup had received either haloperidol or placebo. The resulting four posterior distributions were then used to calculate:

– Absolute differences within each motor subgroup (MDs/RDs).
– Probabilities of benefit or harm in each motor subgroup defined as MD > 0/RD < 1 or MD < 0/RD > 1. For the outcome *days with rescue medications* any benefit was defined as MD < 0 and any harm as MD > 0.
– Differences in intervention effects between motor subgroups (difference between MDs/RDs).
– Probabilities of interaction in either direction (e.g., larger positive effect in hyperactive motor subtype or larger positive effect in hypoactive motor subtype)

Posteriors were summarised using median values as point estimates and 95% percentile‐based CrIs. As missing outcome data were limited (4.5% or less), complete case analyses were conducted. There were no missing data for stratification variables. Due to the post hoc nature of the study, all results should be interpreted cautiously.

## 3 Results

Amongst the 987 participants, 540 (54.7%) had hypoactive delirium and 447 (45.3%) had hyperactive delirium at randomisation. Baseline characteristics were generally well balanced between intervention groups. However, participants with hypoactive delirium were less frequently male, had more use of renal‐replacement therapy and had more days from ICU admission to randomisation compared with those with hyperactive delirium (Table 1). Most participants with hypoactive delirium at randomisation remained hypoactive, whereas participants with hyperactive delirium often transitioned to mixed delirium, experiencing both hyperactive and hypoactive episodes during their ICU stay (Figure S6).

**TABLE 1:** Baseline characteristics stratified by delirium motor subtype at randomisation.

Covariate	Hyperactive delirium		Hypoactive delirium	
	Haloperidol	Placebo	Haloperidol	Placebo
	*N* = 224	*N* = 223	*N* = 277	*N* = 263
Age, median [IQR]	69.0 [60.0–76.0]	70.0 [63.0–76.0]	71.0 [63.0–77.0]	72.0 [63.5–77.0]
Female sex, *N* (%)	62 (27.7)	57 (25.6)	115 (41.5)	104 (39.5)
Risk factors for delirium				
History of traumatic brain injury with (< 6 months), *N* (%)	4 (1.8)	7 (3.1)	4 (1.4)	0 (0.0)
History of stroke (< 6 months), *N* (%)	4 (1.8)	5 (2.2)	8 (2.9)	12 (4.6)
History of mental illness (< 6 months), *N* (%)	10 (4.5)	13 (5.8)	19 (6.9)	16 (6.1)
Smoking, *N* (%)	84 (37.5)	64 (28.7)	72 (26.0)	83 (31.6)
Alcohol overconsumption, *N* (%)	39 (17.4)	36 (16.1)	46 (16.6)	41 (15.6)
Habitual use of benzodiazepines, *N* (%)	7 (3.1)	8 (3.6)	6 (2.2)	9 (3.4)
Treatment with benzodiazepines before randomisation, *N* (%)	81 (36.2)	69 (30.9)	85 (30.7)	74 (28.1)
Co‐existing conditions				
Hematologic cancer, *N* (%)	13 (5.8)	17 (7.6)	20 (7.2)	14 (5.3)
Metastatic cancer, *N* (%)	7 (3.1)	7 (3.1)	8 (2.9)	8 (3.0)
COVID‐19 infection, *N* (%)	15 (6.7)	27 (12.1)	19 (6.9)	26 (9.9)
Admission type				
Surgical admission, *N* (%)	81 (36.2)	66 (29.6)	102 (36.8)	87 (33.1)
Medical admission, *N* (%)	143 (63.8)	157 (70.4)	175 (63.2)	176 (66.9)
Use of organ support at randomisation				
Respiratory support,^a^ *N* (%)	135 (60.3)	138 (61.9)	185 (66.8)	167 (63.5)
Vasopressors/inotropes,^b^ *N* (%)	119 (53.1)	111 (49.8)	153 (55.2)	128 (48.7)
Renal‐replacement therapy,^c^ *N* (%)	31 (13.8)	28 (12.6)	46 (16.6)	42 (16.0)
Simplified Mortality Score for the Intensive Care Unit (SMS‐ICU),^d^ median [IQR]	19 [16–22]	20.0 [16–24]	20.0 [17–24]	20.0 [16–24]
Days from hospital to ICU admission, median [IQR]	1.0 [0.0–5.0]	2.0 [0.0–5.0]	1.0 [0.0–5.0]	1.0 [0.0–4.0]
Days from ICU admission to randomisation, median [IQR]	3.6 [1.4–7.5]	3.6 [1.4–7.5]	5.0 [1.9–11.9]	4.4 [2.0–10.4]

*Note:* Definitions of all baseline variables are available in the primary publication [11] and protocol [16].

^a^ Mechanical ventilation was defined as invasive or non‐invasive mechanical ventilation, including use of a continuous positive airway pressure (CPAP) mask or CPAP by means of tracheostomy within 24 h before randomisation. Intermittent CPAP was not defined as mechanical ventilation.

^b^ Use of vasopressors or inotropes was defined as continuous infusion of norepinephrine, epinephrine, phenylephrine, vasopressin analogues, dopamine, dobutamine, milrinone or levosimendan within the 24 h before randomisation.

^c^ Renal‐replacement therapy was defined as short‐term or prolonged intermittent or continuous renal‐replacement therapy within 24 h before randomisation.

^d^ The Simplified Mortality Score for the Intensive Care Unit (SMS‐ICU) [26] is a severity score that ranges from 0 to 42 (corresponding to a predicted 90‐day mortality range of 3.3% to 91.0%).

**FIGURE 1:**
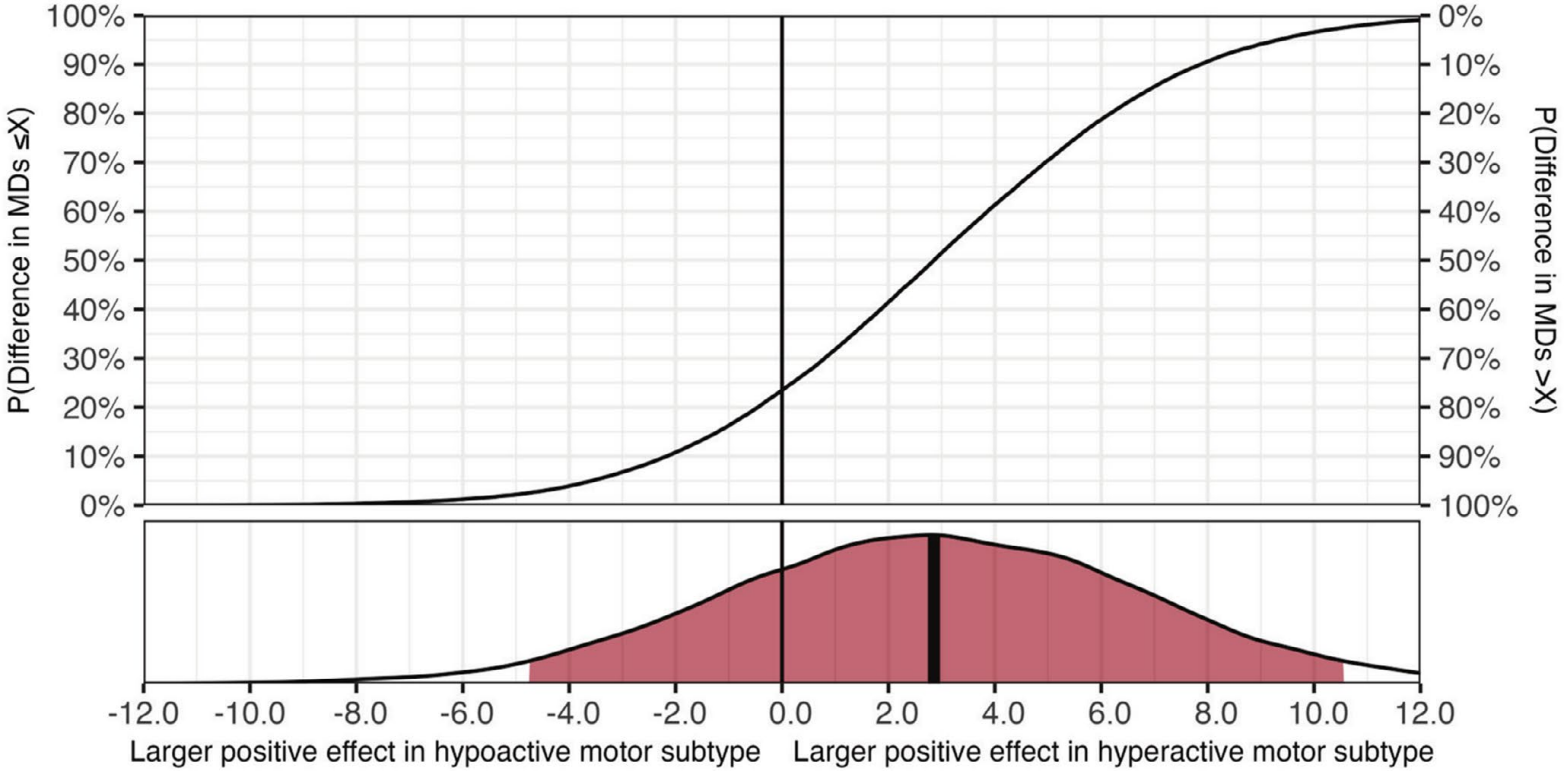
Days alive and out of hospital. The full posterior probability distribution for differences in the effect of haloperidol treatment between hyperactive and hypoactive delirium on days alive and out of hospital. These results are adjusted for stratification variables (trial site and delirium motor subtype) and include an interaction term between intervention and delirium motor subtype. A difference in MDs > 0 indicates a larger positive effect of haloperidol treatment in hyperactive delirium compared with hypoactive delirium. The lower subplots display the full posterior distribution, with the bold vertical line representing the median value (used as the point estimate) and the shaded area in red representing the percentile‐based 95% credible interval. The upper subplots display the cumulative posterior distributions and thereby the probabilities of different effect sizes (*X*‐axis). The left *Y*‐axis represents the probability that is less than or equal to a given value, whilst the right *Y*‐axis represents the probability that the effect is more than a given value. The vertical black line represents no difference in intervention effect between delirium subgroups.

For both motor subgroups, haloperidol was associated with more days alive and out of hospital compared with placebo (Table 2). The probabilities of having any benefit of haloperidol were 72.8% and 93.2% for hypoactive delirium and hyperactive delirium, respectively. We found a 76.5% probability that haloperidol was associated with a larger effect in participants with hyperactive delirium compared with hypoactive delirium, estimated as a difference in MDs of 2.9 (95% CrI −4.8 to 10.6) days more in participants with hyperactive delirium (Figure 1).

**TABLE 2:** Outcomes.

Outcome	Haloperidol	Placebo	Absolute difference: mean difference or risk difference (95% CrI)	Probability of any benefit with haloperidol	Probability of any harm with haloperidol
Days alive and out of hospital					
Hyperactive, median [IQR]	46 [0.0–72.0]	36.5 [0.0–70.3]	MD: 4.5 days (95% CrI: −1.4 to 10.4)	93.2%	6.8%
Hypoactive, median [IQR]	33.5 [0.0–66.0]	27.5 [0.0–66.0]	MD 1.6 days (95% CrI: −3.6 to 6.9)	72.8%	27.2%
Difference in intervention effects with haloperidol between delirium motor subtypes			Difference in MDs: 2.9 days (95% CrI: −4.8 to 10.6)	Probability of larger positive effect with haloperidol in hyperactive subgroup: 76.5%	Probability of larger positive effect with haloperidol in hypoactive subgroup: 23.5%
90‐day mortality					
Hyperactive, no. (%)	74 (33.0%)	94 (42.2%)	RD: −8.8%‐points (95% CrI: −17.5%‐points to −0.2%‐points)	97.8%	2.2%
Hypoactive, no. (%)	108 (39.0%)	116 (44.3%)	RD: −5.2%‐points (95% CrI: −13.1%‐points to 2.8%‐points)	89.9%	10.1%
Difference in intervention effects with haloperidol between delirium motor subtypes			Difference in RDs: −3.7%‐points (95% CrI: −15.3%‐points to 7.8%‐points)	Probability of larger positive effect with haloperidol in hyperactive subgroup: 73.4%	Probability of larger positive effect with haloperidol in hypoactive subgroup: 26.6%
Days alive without delirium or coma					
Hyperactive	83.0 [17.5–87.0]	80.0 [6.0–87.0]	MD: 6.7 days (95% CrI: 0.0 to 13.5) days	97.5%	2.5%
Hypoactive	78.0 [8.0–87.0]	76.0 [2.2–86.0]	MD: 3.6 days (95% CrI: −2.6 to 9.7) days	87.4%	12.6%
Difference in intervention effects between delirium motor subtypes			Difference in MDs: 3.1 days (95% CrI: −5.8 to 11.9) days	Probability of larger positive effect with haloperidol in hyperactive subgroup: 75.4%	Probability of larger positive effect with haloperidol in hypoactive subgroup: 24.6%
Days alive without mechanical ventilation					
Hyperactive	84.0 [10.8–89.0]	81.0 [9.0–89.0]	MD: 5.1 days (95% CrI: −1.6 to 11.9) days	93.2%	6.9%
Hypoactive	77.0 [10.0–88.0]	75.0 [6.0–88.5]	MD: 3.0 days (95% CrI: −3.0 to 9.0) days	83.8%	16.2%
Difference in intervention effects between delirium motor subtypes			Difference in MDs: 2.1 days (95% CrI: −6.6 to 10.8) days	Probability of larger positive effect with haloperidol in hyperactive subgroup: 68.1%	Probability of larger positive effect with haloperidol in hypoactive subgroup: 31.9%
One or more SARs^a^					
Hyperactive	5 (2.2)	4 (1.8)	RD: 0.3%‐points (95% CrI: −1.8%‐points to 2.7%‐points)	37.5%	62.5%
Hypoactive	6 (2.2)	5 (1.9)	RD: 0.2%‐points (95% CrI: −1.6%‐points to 2.1%‐points)	39.6%	60.4%
Difference in intervention effects between delirium motor subtypes			Difference in RDs: 0.1%‐points (95% CrI: −2.3%‐points to 2.6%‐points)	Probability of larger positive effect with haloperidol in hyperactive subgroup: 46.2%	Probability of larger positive effect with haloperidol in hypoactive subgroup: 53.8%
Number of patients using rescue medications^b^					
Hyperactive	160 (71.4)	175 (78.5)	RD: −6.0% (95% CrI: −13.7% to 1.7%)	93.6%	6.4%
Hypoactive	128 (46.2)	127 (48.3)	RD: −2.4% (95% CrI: −10.4% to 5.6%)	71.7%	28.3%
Difference in intervention effects between delirium motor subtypes			Difference in RDs: −3.6% (95% CrI: −14.5% to 7.4%)	Probability of larger positive effect with haloperidol in hyperactive subgroup: 74.0%	Probability of larger positive effect with haloperidol in hypoactive subgroup: 26.0%
Days with rescue medication					
Hyperactive	2.0 [0.0–4.0]	2.0 [1.0–4.5]	MD: 0.3 (95% CrI: −0.6 to 1.1) days	27.5%	72.5%
Hypoactive	0.0 [0.0–3.0]	0.0 [0.0–3.0]	MD: −0.1 (95% CrI: −0.9 to 0.7) days	58.2%	41.8%
Difference in intervention effects between delirium motor subtypes			Difference in MDs: 0.4 (95% CrI: −0.8 to 1.5) days	Probability of larger positive effect with haloperidol in hyperactive subgroup: 28.1%	Probability of larger positive effect with haloperidol in hypoactive subgroup: 71.9%

*Note:* Outcome data stratified by intervention and delirium motor subtype are raw descriptive data. The effect estimates are presented as sample‐average treatment effects with median posterior values as point estimates and percentile‐based 95% credible intervals (CrIs). All analyses were adjusted for stratification variables being trial site and delirium motor subtype, and in addition, included interaction terms between delirium motor subtype and the intervention effect. Any benefit is the probability of an adjusted MD > 0 days or an adjusted RD < 0%‐points, except for the outcome *days with rescue medication*, where any benefit is the probability of an adjusted MD < 0 days. Differences in intervention effects between delirium motor subtypes (hyperactive versus hypoactive) are presented for each outcome with corresponding probabilities of larger positive effects with haloperidol in hyperactive delirium (difference in MDs > 0/RDs < 1) or probabilities of larger positive effects with haloperidol in hypoactive delirium (difference in MDs < 0/RDs > 1).

^a^ Serious adverse reactions (SARs) included the following: anaphylactic reactions, agranulocytosis, pancytopenia, ventricular arrhythmia, extrapyramidal symptoms, tardive dyskinesia, malignant neuroleptic syndrome and acute hepatic failure.

^b^ Rescue medications are defined as use of benzodiazepines, propofol or alpha‐2 agonists to treat delirium.

**FIGURE 2:**
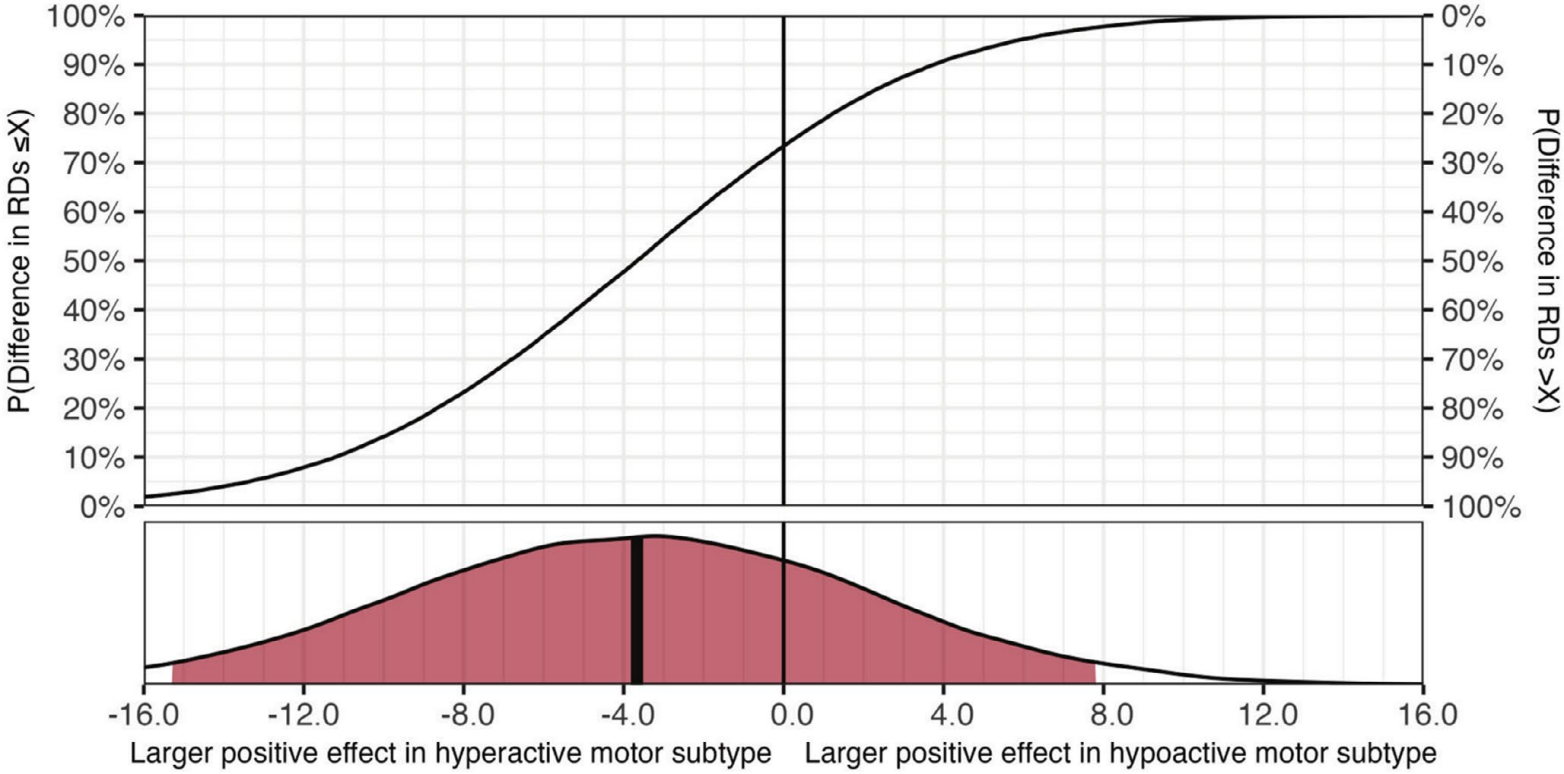
Mortality. The full posterior probability distribution for differences in the effect of haloperidol treatment between hyperactive and hypoactive delirium on 90‐day mortality is presented. These results are adjusted for stratification variables (trial site and delirium motor subtype) and include an interaction term between intervention and delirium motor subtype. A difference in RDs < 0 indicates a larger positive effect of haloperidol treatment in hyperactive delirium compared with hypoactive delirium. The lower subplots display the full posterior distribution, with the bold vertical line representing the median value (used as the point estimate) and the shaded area in red representing the percentile‐based 95% credible interval. The upper subplots display the cumulative posterior distributions and thereby the probabilities of different effect sizes (*X*‐axis). The left *Y*‐axis represents the probability that is less than or equal to a given value, whilst the right Y‐axis represents the probability that the effect is more than a given value. The vertical black line represents no difference in intervention effect between delirium subgroups.

We found that haloperidol treatment was associated with lower probabilities of mortality for both subgroups; RD −8.8%‐points (95% CrI: −17.5 to −0.2%‐points) for hyperactive delirium and RD −5.2%‐points (95% CrI: −13.1 to 2.8%‐points) for hypoactive delirium. The effect of haloperidol seemed higher amongst participants with hyperactive delirium calculated as a difference between RDs of −3.7%‐points (95% CrI: −15.3 to 7.8%‐points); the probability was 73.4% for a larger positive effect of haloperidol in hyperactive delirium compared with hypoactive delirium (Figure 2).

Similar results were obtained for the outcomes days alive without delirium or coma, days alive without mechanical ventilation and use of rescue medications (Table 2). For both subgroups, haloperidol treatment was associated with more days alive without delirium or coma and mechanical ventilation, and fewer patients received rescue medications. The probabilities of finding a larger positive effect of haloperidol in participants with hyperactive delirium were 75.4% for days alive without delirium or coma, 68.1% for days alive without mechanical ventilation and 74.0% for the use of rescue medications (Table 2; Figures S1, S2 and S4).

We found no clinically relevant differences between intervention groups or subgroups when assessing SARs or days with rescue medication as differences were small and had narrow credible intervals including both benefit and harm (Table 2; Figures S3 and S5).

All model diagnostics were considered acceptable (Supporting Information S1: Technical details and model diagnostics).

## 4 Discussion

In this post hoc study investigating the effects of haloperidol on hypoactive and hyperactive delirium, we found moderate to high probabilities (72%–98%) that haloperidol treatment was associated with beneficial effects across both subgroups in terms of more days alive and out of hospital, days without delirium or coma, days without mechanical ventilation, less need for rescue medication and lower mortality.

Whilst probabilities of beneficial effects were moderate to high in both subgroups, the results were most compatible with larger positive effects of haloperidol in participants with hyperactive delirium than in those with hypoactive delirium, with probabilities ranging from 68% to 75%.

The direction of the effect of haloperidol appeared consistent across both delirium motor subtypes, although with a potentially greater benefit in hyperactive delirium for most outcomes. The pathophysiological mechanism underlying this difference in response remains unclear. Traditionally, delirium motor subtypes have been linked to dopaminergic activity, with hypoactivity associated with hypodopaminergic states—similar to bradykinesia in Parkinson's disease—and hyperactivity linked to increased dopamine levels, as seen with agitation induced by pro‐dopaminergic drugs [27]. However, dopamine's role in delirium is complex, as dopamine blockage can induce both akathisia and bradykinesia [28]. Haloperidol exerts its primary antipsychotic effect through strong antagonism of dopamine D2 receptors, which influences both motor and behavioural pathways. Additionally, it has antagonistic effects on dopamine D3 and D4 receptors, serotonin 5‐HT2A receptors and α1‐adrenergic receptors, which may contribute to its sedative properties [9]. These broader pharmacodynamic effects may explain why haloperidol's impact is not strictly limited to hyperactive delirium.

This study focused on the effects of haloperidol on hyperactive and hypoactive delirium at randomisation, but motor subtypes are inherently dynamic. However, whilst some participants transitioned between subtypes during the trial, such post‐randomisation shifts could be influenced by the intervention itself. Consequently, analysing the effect of haloperidol on mixed delirium could introduce bias and at least hamper interpretability. But if there is a natural shift in motoric subtypes during the course of delirium, this study design cannot account for such changes as it only includes motoric subtypes at randomisation. Our data (Figure S6) indicate that the transition between motoric subtypes during ICU stay was similar across intervention groups and baseline delirium subtypes. Our findings contribute to the growing interest in delirium subtyping, whether based on motoric presentation, phenotypic characteristics, endotypes or underlying pathophysiological mechanisms. Whilst haloperidol demonstrated a similar directional effect in both subtypes, the result suggests a potentially greater benefit in participants with hyperactive delirium, indicating possible quantitative heterogeneity in treatment effect across motoric subtypes. These findings support ongoing efforts to refine delirium subtyping by demonstrating potential heterogeneity in treatment response across motor subtypes, as highlighted by recent initiatives to improve phenotypic and pathophysiological classification [8].

The MIND‐USA trial also investigated haloperidol's effect in ICU patients with delirium in a three‐armed trial (ziprasidone, haloperidol and placebo). In that trial, the majority (90%) of participants had hypoactive delirium at baseline, and no data on the heterogeneity of treatment effects across delirium motor subtypes (at baseline) were available [29]. This limits direct comparison with our findings but underlines the relevance of future trials considering differential effects based on motor subtypes.

The primary strength of this study is the robust design of the AID‐ICU trial, which included a moderately large sample size, blinding, placebo‐controlled design and a high level of data completeness [11]. Additionally, the study adhered to a statistical analysis plan, which was publicly registered before conducting the analyses (but written after the primary trial results were known), ensuring transparency. However, several limitations should be acknowledged. As a post hoc analysis, all findings should be interpreted as exploratory, as the primary results of the trial were available when designing this study. Furthermore, delirium motor subtypes were classified based on clinical assessment or the Richmond Agitation‐Sedation Scale (RASS) [30] at the time of delirium screening at randomisation, without the use of a formal subtyping scheme. Whilst this approach reflects clinical practice, it introduces a risk of misclassification. However, given the distinct clinical characteristics of hypoactive and hyperactive delirium, misclassification is likely minimal. Lastly, despite consistent results in treatment effects across subgroups and outcomes, the precision of our estimates remains limited, and the observed probabilities were mostly moderately high, implying remaining uncertainty. This is reflected in the wide credible intervals, which encompass both potential benefits and harms, highlighting the uncertainty of the findings.

## 5 Conclusions

In this exploratory study, compared with placebo, haloperidol was associated with moderate to high probabilities of benefit in both hypoactive and hyperactive delirium, with moderate probabilities of greater effects in hyperactive delirium. However, substantial uncertainty remains, and we cannot exclude either harm or benefit of either intervention in either subgroup; therefore further research is warranted.

## Author Contributions

**Nina C. Andersen‐Ranberg** and **Anders Granholm:** wrote the protocol. **Nina C. Andersen‐Ranberg** and **Anders Granholm:** conducted and supervised the statistical analyses. **Nina C. Andersen‐Ranberg:** drafted the manuscript. All authors reviewed and approved the manuscript for final publication.

## Funding

This work was supported by Innovation Fund Denmark (Grant No. 4108‐00011B), Regional Medicines Fund (Grant No. R124‐2651), Zealand Region Research Fund, Grosserer L.F. Foghts Fond, and Hindsgavl Intensiv Symposium. The funding had no influence on the trial.

## Ethics Statement

The AID‐ICU trial was conducted in accordance with the ethical standards of the 1964 Helsinki Declaration and its later amendments. Ethical approval was obtained from the Danish Health Ethics Committee, reference number SJ‐646.

## Consent

Informed consent was obtained from all individual participants included in the study.

## Conflicts of Interest

The Department of Intensive Care at Zealand University Hospital, Køge (N.C.A.‐R., L.M.P. and O.M.) has received funding for other projects unrelated to this work from The Novo Nordisk Foundation and Zealand Region Research Fund.

The Department of Intensive Care at Rigshospitalet (A.P., M.O.C., A.G. and S.E.D.) has received funding for other projects unrelated to this work from Sygeforsikringen ‘danmark’, Den Frie Forskningsfond, and The Novo Nordisk Foundation.

The Department of Anaesthesiology and Intensive Care at Copenhagen University Hospital—North Zealand (M.B.) has received funding for other projects unrelated to this work from Sygeforsikring ‘danmark’ and The Novo Nordisk Foundation.

## Supporting information


**Figure S1:** Days alive without delirium or coma in the ICU.
**Figure S2:** Days alive without mechanical ventilation.
**Figure S3:** Number of patients with one or more serious adverse reactions.
**Figure S4:** Number of patients using rescue medications.
**Figure S5:** Days with rescue medications.
**Figure S6:** Changes in delirium subtypes during ICU stay by intervention group.

## Data Availability

De‐identified data will be made available upon reasonable request, this will be decided by the AID‐ICU steering committee.
