# Supplementary material for: Haloperidol for Hypoactive and Hyperactive Delirium in ICU Patients—A Post Hoc Bayesian Analysis of the AID‐ICU Trial
Source: Acta Anaesthesiol Scand. 2026 Sep 18;70(9):e70336. doi: 10.1111/aas.70336 (PMC13588715; doi:10.1111/aas.70336)
Supplement: Supplementary file 1 [file AAS-70-0-s001.pdf]

Haloperidol for hypoactive and hyperactive delirium in ICU patients – a post-hoc Bayesian analysis of the AID-ICU trial

Electronic Supplementary Material

Table of contents

Enrolment criteria of the AID-ICU trial **2**

Detailed stratification and outcome definitions **4**

Model specifications **7**

Prior specifications **8**

Technical details and model diagnostics **10**

Calculation of effect estimates **10**

Figure S1 - Days alive without delirium or coma in the ICU **12**

Figure S2 – Days alive without mechanical ventilation **13**

Figure S3 – Number of patients with one or more Serious Adverse Reactions **14**

Figure S4 – Number of patients using rescue medications **15**

Figure S5 – Days with rescue medications **16**

Figure S6 – Changes in delirium subtypes during ICU stay by intervention group **17**

STROBE checklist **18**

References **20**

Enrolment criteria of the AID-ICU trial

Inclusion criteria

- Acute admission to the intensive care unit (ICU): a non-planned admission. This does not include planned recovery after surgery or similar planned admissions. ICU admission does not include admissions to semi-intensive care, intermediate intensive care or similar beds.
- Aged ≥ 18 years: the age of the patient in whole years at the time of randomization. The age will be calculated from date of birth.
- Diagnosed delirium: delirium diagnosed by a validated screening tool as either the confusion assessment method for the ICU (CAM-ICU)[1] or Intensive Care Delirium Screening Checklist (ICDSC).[2] A patient is diagnosed with delirium if the CAM-ICU assessment is positive or the ICDSC score is ≥ 4.

Exclusion criteria

- Contraindications to haloperidol: any history of intolerance to haloperidol or additives, known Parkinson disease or other extrapyramidal symptoms, known QTc prolongation, history of tardive dyskinesia, history of ventricular arrythmia or torsades de pointes, uncorrected hypokalaemia (a potassium level needing action judged by clinician, only if not corrected), non-pharmacological coma. Coma is defined by the following scales of level of consciousness: Richmond agitation-sedation scale (RASS) -4 to -5[3], Ramsey sedation score (RMS) 4-6[4], Motoric activity assessment scale (MAAS) 1-0, or Glasgow Coma Scale < 8 (GCS), Reaction Level Scale < 3 (RLS), Riker Sedation Agitation Scale (SAS) 1-2. Further, RASS -3 may be considered as coma if this is the judgement of the treating physician.
- Habitual treatment with antipsychotic medication: Daily intake or use of prolonged release medication (any form) of any antipsychotic with the ATC code N05A, which includes the following drugs:
    - Typical antipsychotics: chlorprothixene, flupentixol, haloperidol, levomepromazine, loxapine, melperone, perphenazine, periciazine, pimozide, prochlorperazine, zuclopenthixol, pipamperone, sulpiride
    - Atypical antipsychotics: amisulpride, aripiprazole, asenapine, clozapine, lurasidone, olanzapine, paliperidone, quetiapine, risperidone, sertindole, ziprasidone

- Treatment with antipsychotics in the ICU prior to inclusion: if the patient has been treated with antipsychotics (above mentioned) in the ICU before inclusion. Antipsychotic treatment (not habitual) in the general ward (e.g., due to delirium) prior to ICU admittance is accepted. However, the antipsychotic treatment should be discontinued when the patient is admitted to the ICU.
- Permanently incompetent patient: a patient who permanently is unable to make decisions about his/her affairs (e.g., memory disorder, mental retardation). Patients may or may not have a legal guardian. The attending physician makes this assessment.
- Delirium assessment non applicable: this includes language barriers (patients with foreign language where delirium assessment cannot be confidently performed by the site staff), patients who are deaf, blind or aphasic. Comatose patients are not applicable for delirium assessment. Coma is defined by the following scales of level of consciousness: RASS -4 to -5[3], Ramsey sedation score 4-6[4], MAAS 1- 0[5], GCS < 8[6], SAS 1-2[7]. Further, RASS -3 may be considered as coma if this is the judgement of the treating physician. If a patient's coma is considered related to administration of sedative agents, an effort should be made to reduce or terminate the sedative treatment, according to the clinician's discretion.
- Withdrawal from active therapy or brain death: patients where withdrawal or brain death is documented in the patient's charts.
- Known pregnancy: fertile women with positive urine test for human chorionic gonadotropin (hCG) or plasma-hCG.
- Consent not obtainable according to national regulations: patients where the clinician or investigator is unable to obtain necessary consent before inclusion of the patient according to the national regulations.
- Patients under involuntary hospitalization (coercive measures) by regulatory authorities.
- Alcohol induced delirium/delirium tremens (ICD10: F10.4x): Delirium caused by withdrawal of alcohol after persistent alcohol use. The withdrawal may be complicated by convulsions.

Detailed stratification and outcome definitions

Stratification variables:

- *Site:* defined as the ICU where the patient was randomized. Sites that recruited less than 10 patients are merged into one aggregate site.
- *Delirium motor subtype at randomisation*: delirium motor subtype is either hypoactive or hyperactive. This should reflect how the patient is clinically described at the time of randomisation. The motor subtypes are described below:
    - Hypoactive: if the patient is described as hypoactive and is positive for delirium. Lying still with open eyes and no clear contact (GCS > 7[6]).
    - Hyperactive: If the patient is described hyperactive and is positive for delirium. Agitated and non-cooperative (pulling tubes and catheters).

Outcomes:

- *Days alive and out of the hospital*: calculated as the number of days the patient is alive and discharged from the hospital within the 90-day intervention period.*
- *90-day mortality*: death from any cause within 90 days post-randomisation.*
- *Days alive without delirium or coma*: total number of days without delirium or coma in the ICU within the 90-day intervention period. Delirium is defined as a positive screening with CAM-ICU or a ICDSC > 4 points. Coma is defined by the following scales of level of consciousness: RASS -4 to -5[3], Ramsey sedation score 4-6[4], MAAS 1- 0[5], GCS < 8[6], SAS 1-2[7]. Further, RASS -3 may be considered as coma if this is the judgement of the treating physician.*
- *Days alive without mechanical ventilation*: total number of days alive without mechanical ventilation within the 90-day intervention period. Mechanical ventilation is defined as invasive or non-invasive mechanical ventilation including continuous positive airway pressure (CPAP) mask or CPAP via tracheostomy. Intermittent CPAP is NOT mechanical ventilation. *
- *Number of patients using rescue medication*: number of patients that were treated with rescue medication in the ICU within the 90-day intervention period. Rescue medication is

defined as the use of propofol, α2-agonists or benzodiazepines to treat uncontrollable delirium (e.g., agitation, insomnia). Administration of these agents for other purposes are not registered as rescue medication. Rescue medication is acknowledged as administration of these agents (propofol, α2-agonist, benzodiazepines) in an 'as needed' formula, bolus or increased infusion rate of ongoing infusions of these agents.

- *Days with rescue medication per patient*: total number of days receiving one or more rescue medications (propofol, benzodiazepines and/or alfa2-agonists) in the ICU within the 90-day intervention period.
- *Number of patients with one or more serious adverse reactions (SARs) to haloperidol to day 90*: patients experiencing one or more SARs to haloperidol within the 90-day intervention period. A SAR is defined as any adverse reaction that results in death, is life threatening, requires hospitalization or prolongation of existing hospitalization, or results in persistent or significant disability or incapacity. An adverse reaction is any undesirable and unintended medical response related to the intervention occurring to a participant during a clinical trial. SARs deemed related to haloperidol are the following:
    - Anaphylactic reactions: defined as urticaria and at least one of the following:
        - Worsened circulation (>20% decrease in blood pressure or >20% increase in vasopressor dose).
        - Increased airway resistance (>20% increase in the peak pressure on the ventilation).
        - Clinical stridor or bronchospasm.
        - Subsequent treatment with bronchodilators.
    - Agranulocytosis: defined as any new drop in granulocytes to $< 0.5 \times 10^9$/l.
    - Pancytopenia: defined as any new drop in red blood cells (as severe anaemia, b-Hgb < 4.3mM (70g/dL), white blood cells ($< 0.5 \times 10^9$/l) and platelets ($<20 \times 10^9$/l)
    - Ventricular arrhythmia: defined as any first onset of ventricular arrhythmia (except premature ventricular contractions (PVCs)) seen on electrocardiogram (ECG) or continuous cardiac monitoring.
    - Extrapyramidal symptoms (EPS): this includes dystonia (continuous spasm and muscle contractions), akathisia (motor restlessness), parkinsonism (characteristic

symptoms such as rigidity, bradykinesia and tremor). Mild forms of tremor or akathisia are NOT considered a SAR.

- Tardive dyskinesia: defined as rhythmical involuntary movements of tongue, face, mouth or jaw.
- Malignant neuroleptic syndrome: syndrome characterized by hyperpyrexia, muscle rigidity, catatonia, autonomic instability (irregular pulse or blood pressure, tachycardia, sweating, cardiac dysrhythmias).
- Acute hepatic failure: defined as severe hepatic failure as judged by the treating doctor or the investigator.

Of note, it should not be possible to have a SAR in the placebo group as a SAR is defined to be related to the trial drug. However, as the trial is blinded all serious adverse events that could be related to the study drug were reported as SARs and were analysed as such.

*Death within the 90-day intervention period was not penalized to zero.[8] The numbers reflect the actual days alive irrespective of survival status later in the intervention period.

Model specifications

These model specifications are in accordance with the publicly registered protocol and statistical analysis plan.[9]

Count outcomes

All count outcomes were analysed using adjusted (for the stratification variables) Bayesian linear regression models (*family = gaussian()* in *brms*-syntax[10]), including an interaction term between delirium motor subtype (subtype) and intervention, specified as:

bf(days ~ 1 + intervention + subtype + intervention:subtype + site, center = FALSE)

The *bf*-function denotes the model formula with the *center = FALSE* statement, indicating that a standard, non-centered intercept was used, i.e., the intercept corresponds to the number of days for a patient in the control group at the reference site (Zealand University Hospital, Koege) and reference delirium motor subtype (hypoactive delirium). In this formula, *days* refers to the number of days between 0 and 90 days for all count outcomes. *1* refers to the intercept for the mean number of 'days' in the control group (placebo-group), when all stratification variables (site and delirium motor subtype) were set to their reference values being the most common category. intervention refers to the intervention (haloperidol) group effect. *site* and *subtype* refer to the stratification variables site and delirium motor subtype at randomisation. *intervention:subtype* refers to the interaction effect between the intervention and delirium motor subtype, meaning the difference in treatment effect between hypoactive and hyperactive delirium.

Binary outcomes

The binary outcomes were analysed using logistic regression models (*family = bernulli()* in *brms*(REF) syntax), specified as:

bf(outcome ~ 1 + intervention + subtype + intervention:subtype + site, center = FALSE)

Here, *outcome* refers to either mortality, participants with one or more SARs or number of participants using rescue medications.

Additional terms in the formula are as specified above.

Models were assessed as described in the Bayesian analysis of the trial [11] and described below.

Prior specifications

The following priors were used for days alive and out of hospital, days alive without mechanical ventilation and days alive without delirium or coma.

- Intercept: a *normal(mean = 25, standard deviation = 40)* prior, corresponding to the expected result in the control group (placebo group), with all other variables at their reference levels (site = Zealand University Hospital Koege, delirium motor subtype = hypoactive being centred on a mean of 25 days with 95% central probability mass between -53.4 and 103.4 days alive and out of hospital. This range exceeds the valid values (0-90) but conveys minimal information as it includes all possible values.
- Adjustment variables: *normal(0, 15)* priors were used for all adjustment variables corresponding to probability distributions for the adjustment variables centred on 0 and 95% central probability mass between -29.4 and 29.4 days alive and out of hospital.
- Intervention effect: a *normal(0, 15)* prior as described for the adjustment variables above.
- Interaction effect: a *normal(0, 15)* prior as described for the adjustment variables above.

For the outcome *number of days with rescue medication* the following priors were used:

- Intercept: a *normal(1.5, 45)* prior corresponding to a probability distribution centred around 1.5 days and 95% central probability mass between -86.7 days and 89.7 days with rescue medications.
- Adjustment variables: same as for other count outcomes.
- Intervention effect: same as for other count outcomes.
- Interaction effect: same as for other count outcomes

For the binary outcomes; 90-day mortality and number of patients using rescue medications, the following priors were used.

- Intercept: a *normal(-0.41, 1.5)* prior – a normally distributed prior with mean -0.41 and SD 1.5 on the log odds ratio scale. Corresponding to a baseline risk (risk of outcome in control group (placebo group) when all adjustment variables are set to their reference values) with 95% central probability mass between 3.4% and 92.6% and a mean probability of 39.9%.
- Adjustment variables: *normal(0, 1)* priors were used for all adjustment variables, corresponding to probability distributions for the effect on the odds ratio scale centred on 1.00 (no difference) and with 95% probability mass between 0.14 and 7.10.
- Intervention effect: a *normal(0, 1)* prior was used as described for the adjustment variables above. This is a weakly informative prior.
- Interaction effect: a *normal(0, 1)* prior was used as described for the adjustment variables above.

For the binary outcome; number of patients with one or more SARs, the following priors were used:

- Intercept: a *normal(-4.0, 1.5)* prior. Corresponding to a baseline risk (percentage of patients experiencing one or more SARs to haloperidol in the control group when all adjustment variables are set to their reference values) with mean probability of 1.8% and 95% central probability mass between 0.1% and 25.7%.
- Adjustment variables: same as described above for other binary outcomes.
- Intervention effect: same as described above for other binary outcomes.
- Interaction effect: same as described above for other binary outcomes.

It is to be noted that it should not be possible to have SARs in the placebo group, as SARs are defined to be related to the trial drug. However, as the trial was blinded all serious adverse events that could be related to the trial drug were reported as SARs.

Technical details and model diagnostics

We used *Stan*'s default dynamic Hamiltonian Monte Carlo sampler.[12] We used 4 chains with 5,000 warm-up iterations and 10,000 post-warm-up iterations each (40,000 posterior samples in total). The bulk/tail effective sample size for the parameter of primary interest (the treatment effect) were required to be at least 5,000 in all models. Chain convergence were assessed by overlain density and trace plots and *Rhat* statistic ≤1.01 were required for all parameters.[13,14] Model fits were further assessed with graphical posterior predictive checks of the group means/proportions[15] and Pareto-smoothed importance sampling leave-one-out cross-validation with focus on the effective number of parameters in the model.[16]

All model diagnostics were considered acceptable, with all criteria mentioned above fulfilled for all models except for the outcomes *number of patients with one or more SARs to haloperidol* and *days with rescue medications*. For these outcomes, the effective number of parameters in the models were substantially lower(SARs 7) or slightly higher (days with escape, 22) than the actual number of parameters (14 for SARs and 15 for days with escape). This may indicate that the models had weak predictive capability, and possible that the models may be misspecified, depending on other diagnostics.[17] Given that all other diagnostics were considered adequate and the relatively low number of days in both groups, these models were considered overall adequate based on all diagnostics.

Calculation of effect estimates

We calculated and report average treatment effects as previously described.[18] The joint posteriors were used to calculate the predicted estimates for all patients in each motor subgroup. Here we *assumed* that they had received either first haloperidol and second placebo independent of their actual group allocation, which corresponds to predicting the actual and counterfactual outcomes for all patients in each motor subgroup. The four posterior distributions of predicted mean values ($hypoactive_{haloperidol}$, $hypoactive_{placebo,}$ $hyperactive_{haloperidol}$, $hyperactive_{placebo}$) were then used to calculate absolute differences in each subgroups ($hypoactive_{haloperidol}$ – $hypoactive_{placebo}$ and $hyperactive_{haloperidol}$ – $hyperactive_{placebo}$) and probabilities of any benefit or harm and probabilities of interactions in either direction (larger positive effect in hyperactive motor subtype or larger positive effect in hypoactive motor subtype).

Any benefit is defined as a difference in MDs > 0 or RDs < 0 for all outcomes except days with escape where any benefit is a difference in MDs < 0. Any harm is a difference in MDs < 0 or RDs > 0 for all outcomes except days with escape where any harm is difference in MDs > 0.

Figure S1 - Days alive without delirium or coma in the ICU

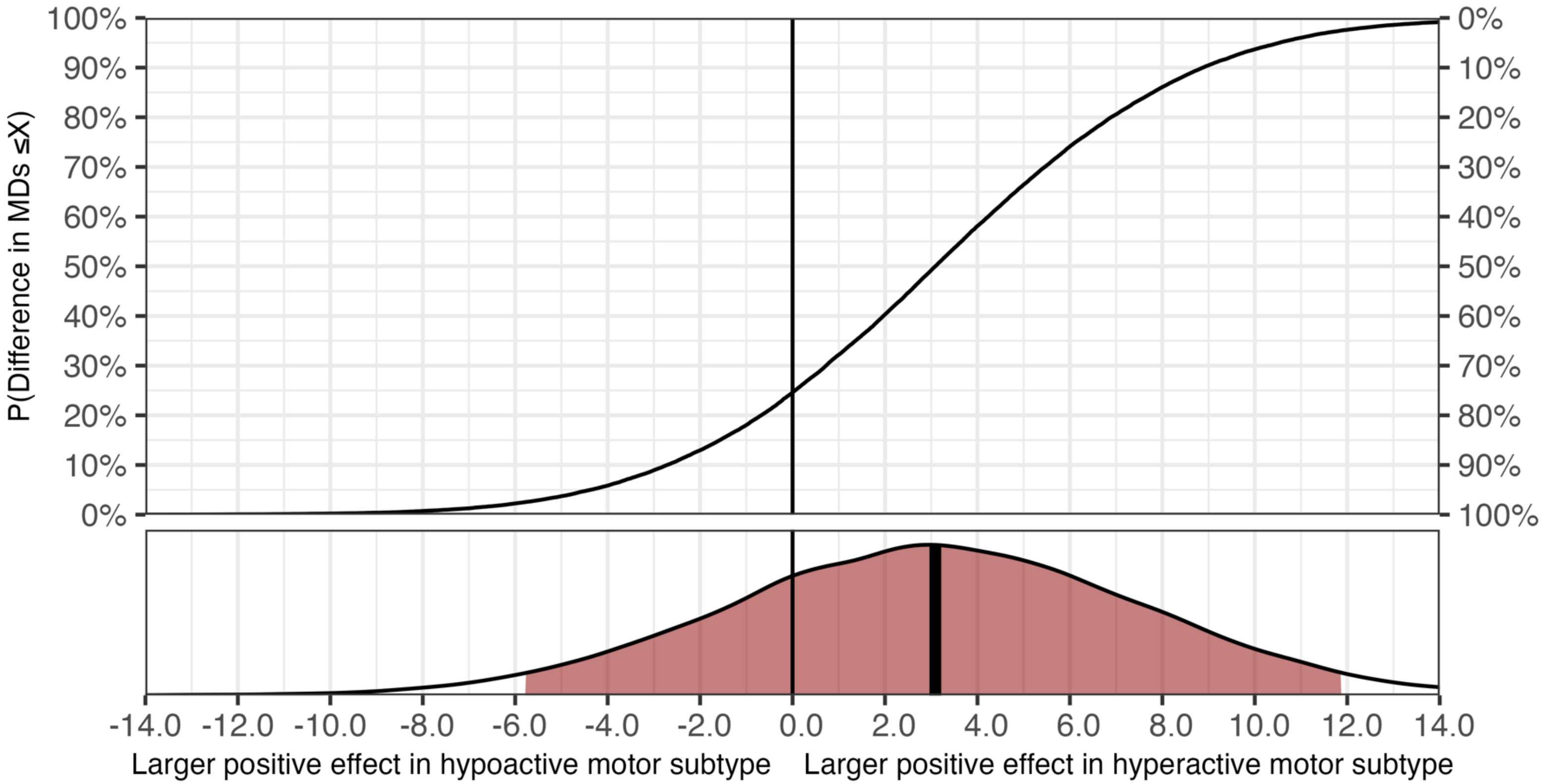

Full posterior probability distribution for the difference in effect of haloperidol treatment between hyperactive and hypoactive delirium on days alive without delirium or coma in the ICU. These results are adjusted for stratification variables (trial site and delirium motor subtype) and include an interaction term between intervention and delirium motor subtype. A difference in MDs > 0 indicate a larger positive effect of haloperidol treatment in hyperactive delirium compared whit hypoactive delirium. The lower subplots display the full posterior distribution, with the bold vertical line representing the median value (used as the point estimate) and the shaded area in red representing the percentile-based 95% credible interval. The upper subplots display the cumulative posterior distributions and thereby the probabilities of different effect sizes (X-axis). The left Y-axis represents the probability that is ≤ given value, while the right Y-axis represents the probability that the effect is > a given value. The vertical black line represents no difference in intervention effect between delirium subgroups.

Figure S2 – Days alive without mechanical ventilation

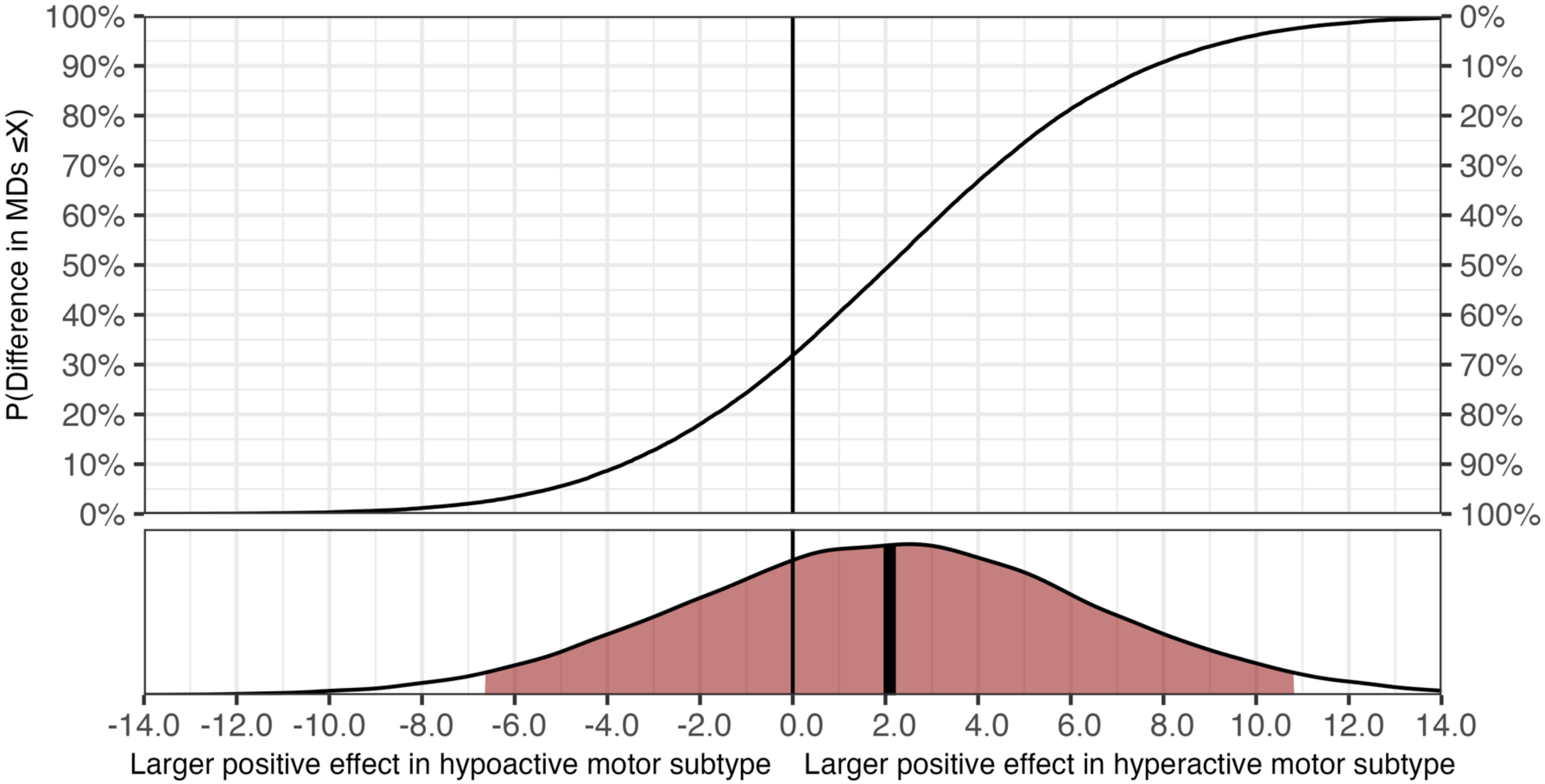

Full posterior probability distribution for the difference in effect of haloperidol treatment between hyperactive and hypoactive delirium on days alive without mechanical ventilation. These results are adjusted for stratification variables (trial site and delirium motor subtype) and include an interaction term between intervention and delirium motor subtype. A difference in MDs > 0 indicate a larger positive effect of haloperidol treatment in hyperactive delirium compared whit hypoactive delirium. The lower subplots display the full posterior distribution, with the bold vertical line representing the median value (used as the point estimate) and the shaded area in red representing the percentile-based 95% credible interval. The upper subplots display the cumulative posterior distributions and thereby the probabilities of different effect sizes (X-axis). The left Y-axis represents the probability that is ≤ given value, while the right Y-axis represents the probability that the effect is > a given value. The vertical black line represents no difference in intervention effect between delirium subgroups.

Figure S3 – Number of patients with one or more Serious Adverse Reactions

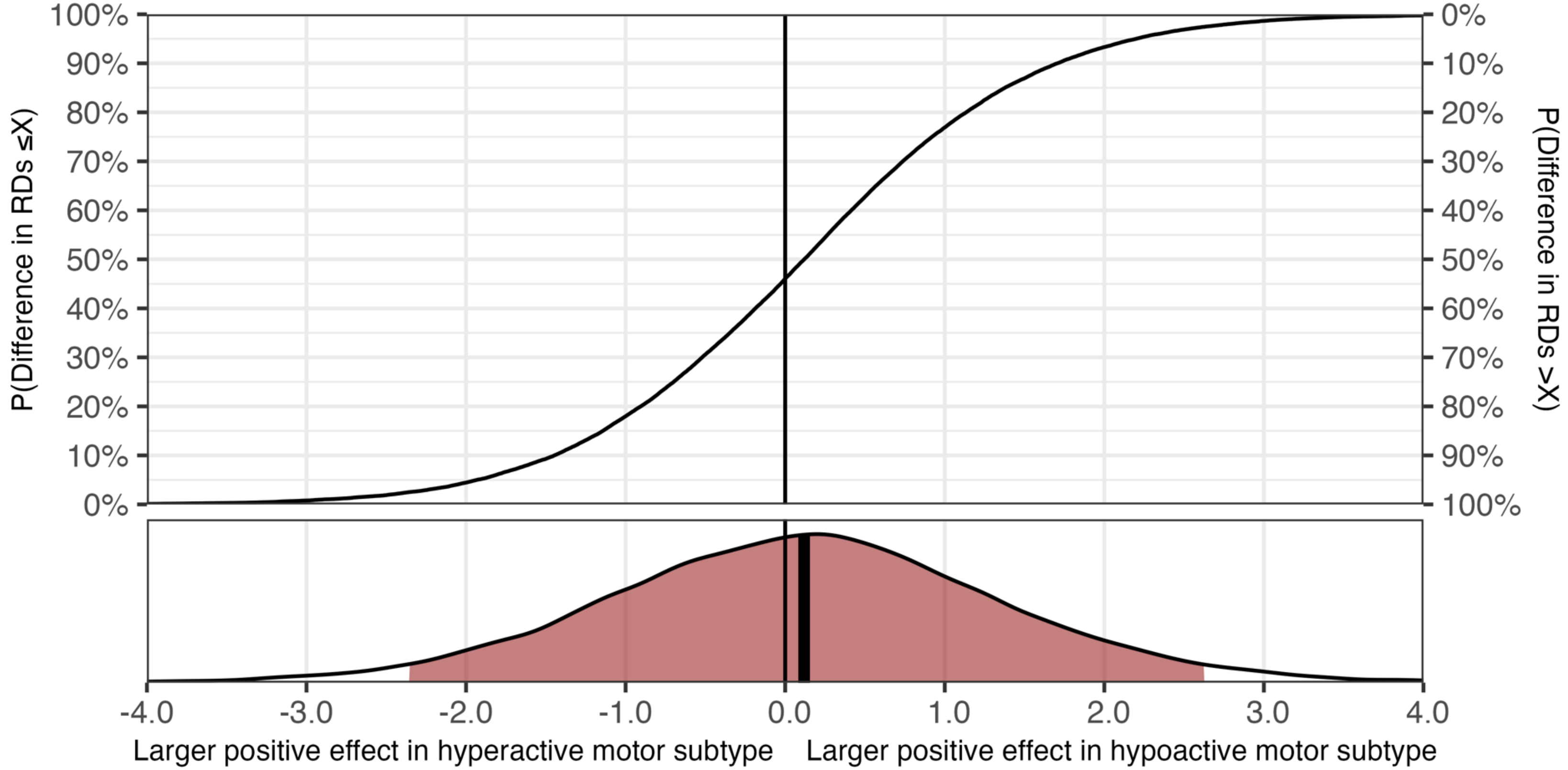

Full posterior probability distribution for the difference in effect of haloperidol treatment between hyperactive and hypoactive delirium on number of patients with one or more Serious Adverse Reactions. These results are adjusted for stratification variables (trial site and delirium motor subtype) and include an interaction term between intervention and delirium motor subtype. A difference in RDs < 0 indicate a larger positive effect of haloperidol treatment in hyperactive delirium compared whit hypoactive delirium. The lower subplots display the full posterior distribution, with the bold vertical line representing the median value (used as the point estimate) and the shaded area in red representing the percentile-based 95% credible interval. The upper subplots display the cumulative posterior distributions and thereby the probabilities of different effect sizes (X-axis). The left Y-axis represents the probability that is ≤ given value, while the right Y-axis represents the probability that the effect is > a given value. The vertical black line represents no difference in intervention effect between delirium subgroups.

Figure S4 – Number of patients using rescue medications

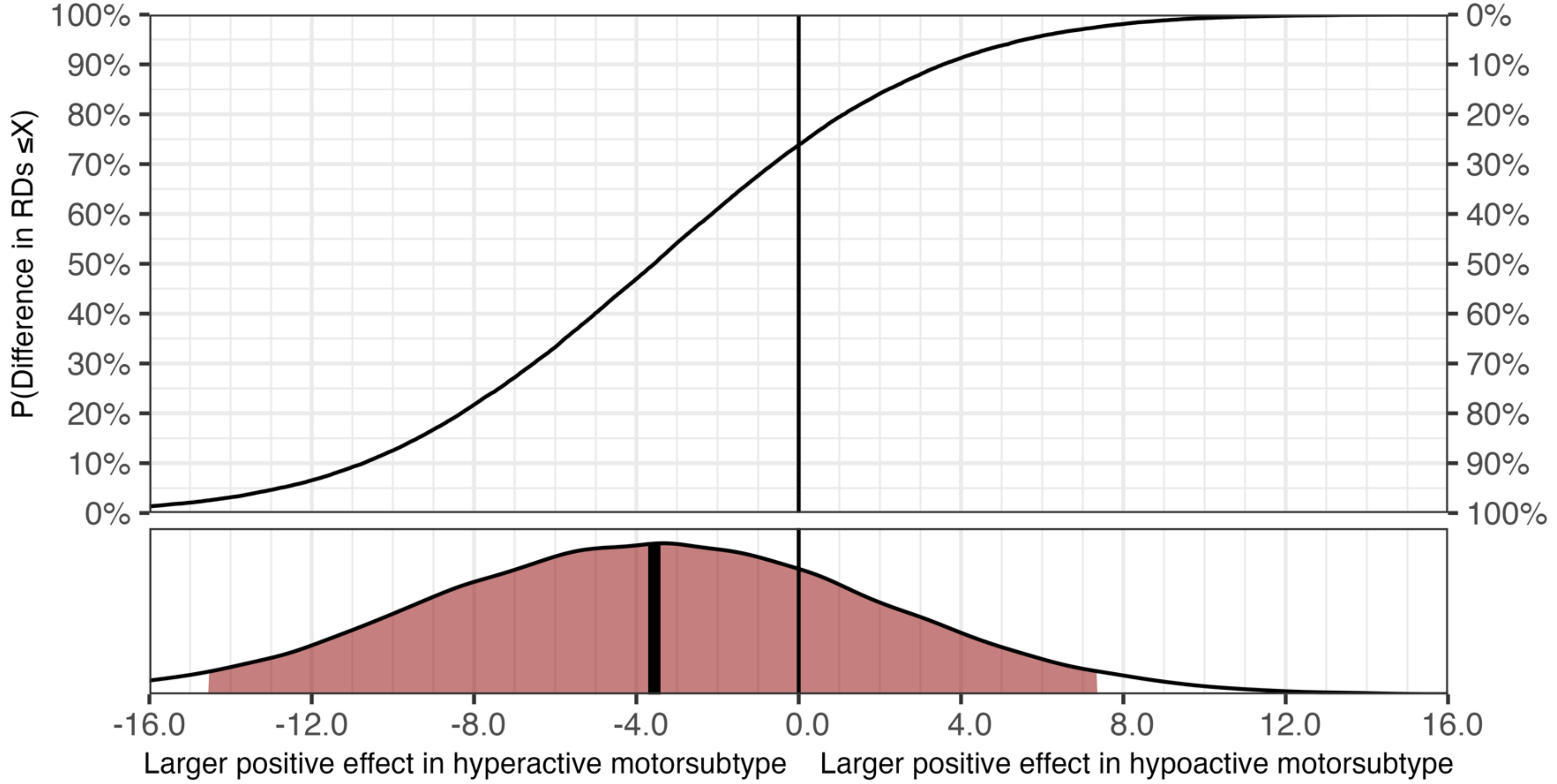

Full posterior probability distribution for the difference in effect of haloperidol treatment between hyperactive and hypoactive delirium on number of patients using rescue medications. These results are adjusted for stratification variables (trial site and delirium motor subtype) and include an interaction term between intervention and delirium motor subtype. A difference in RDs < 0 indicate a larger positive effect of haloperidol treatment in hyperactive delirium compared whit hypoactive delirium. The lower subplots display the full posterior distribution, with the bold vertical line representing the median value (used as the point estimate) and the shaded area in red representing the percentile-based 95% credible interval. The upper subplots display the cumulative posterior distributions and thereby the probabilities of different effect sizes (X-axis). The left Y-axis represents the probability that is ≤ given value, while the right Y-axis represents the probability that the effect is > a given value. The vertical black line represents no difference in intervention effect between delirium subgroups.

Figure S5 – Days with rescue medications

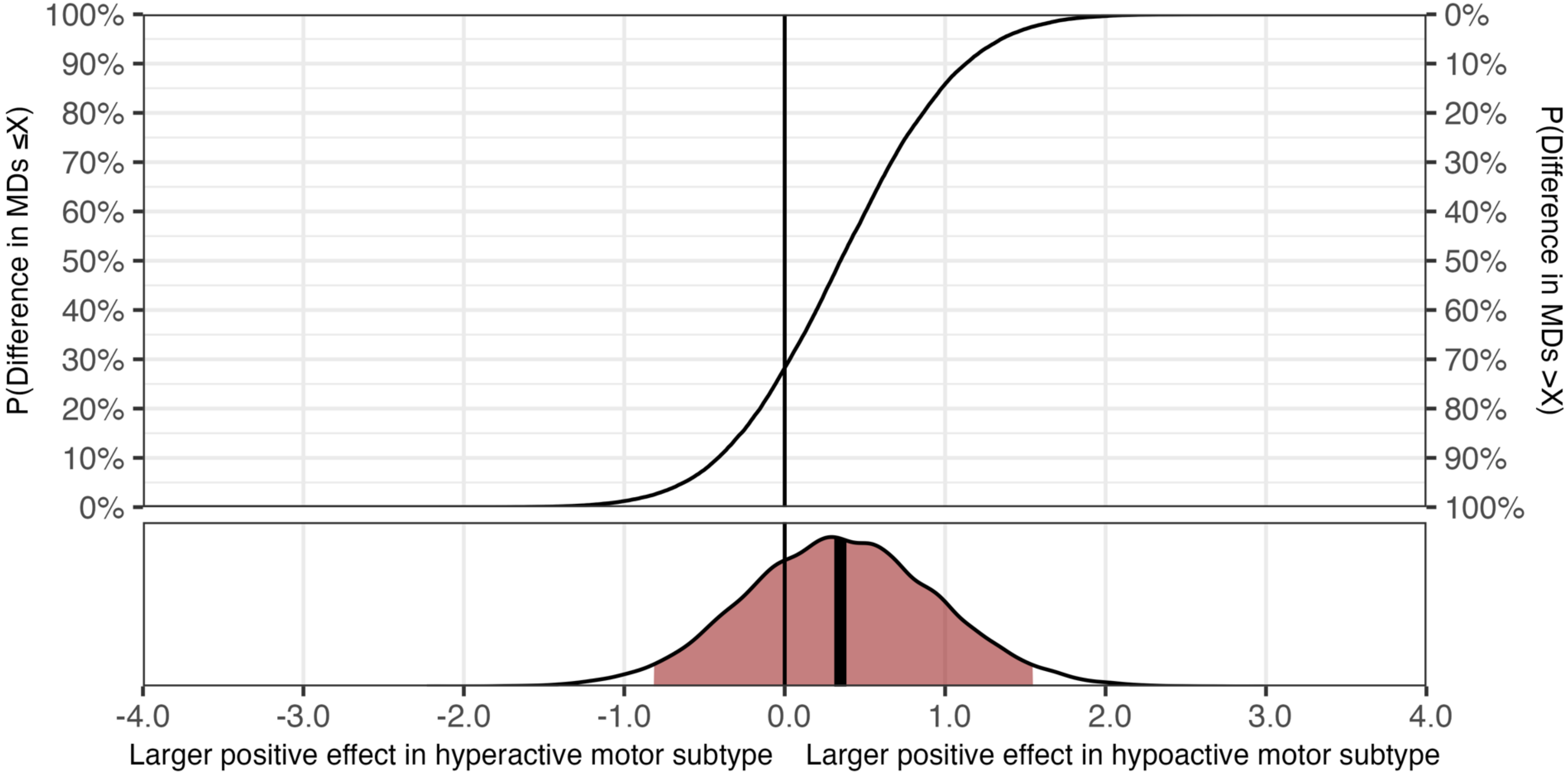

Full posterior probability distribution for the difference in effect of haloperidol treatment between hyperactive and hypoactive delirium on days with rescue medications. These results are adjusted for stratification variables (trial site and delirium motor subtype) and include an interaction term between intervention and delirium motor subtype. A difference in MDs < 0 indicate a larger positive effect of haloperidol treatment in hyperactive delirium compared with hypoactive delirium. The lower subplots display the full posterior distribution, with the bold vertical line representing the median value (used as the point estimate) and the shaded area in red representing the percentile-based 95% credible interval. The upper subplots display the cumulative posterior distributions and thereby the probabilities of different effect sizes (X-axis). The left Y-axis represents the probability that is ≤ given value, while the right Y-axis represents the probability that the effect is > a given value. The vertical black line represents no difference in intervention effect between delirium subgroups.

Figure S6 – Changes in delirium subtypes during ICU stay by intervention group

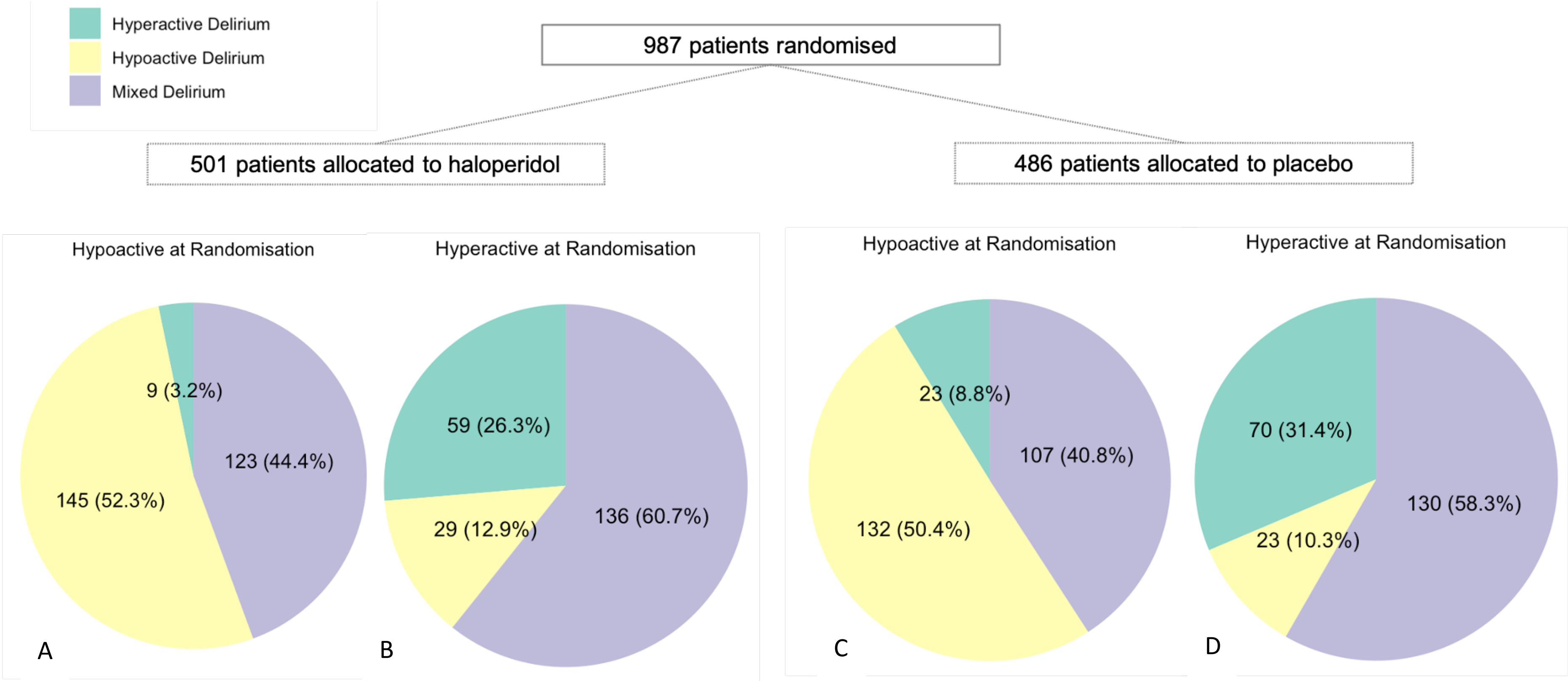

Changes in delirium subtypes during ICU stay by intervention group.
Patients were randomised to receive either haloperidol or placebo, with initial delirium subtypes classified as hyperactive or hypoactive at randomisation. Each pie chart represents the progression of these subtypes throughout the ICU stay. E.g. a patient that had hypoactive delirium at randomisation, but during ICU stay have episodes of both hypoactive and hyperactive delirium will be classified as having mixed delirium in the pie chart. Colors indicate delirium subtypes: yellow for hypoactive**,** green for hyperactive, and purple for mixed delirium. Percentages denote the proportion of patients transitioning to each subtype during their ICU stay.

STROBE checklist

Completed Strengthening the Reporting of Observational Studies in Epidemiology (STROBE) statement checklist for this manuscript. Further information may be found at the AID-ICU trial website (www.cric.nu/aid-icu), the primary trial protocol and statistical analysis plan and the primary trial report of the AID-ICU trial.

Abbreviations: ESM – electronic supplementary material (this file).

Primary trial protocol: AID-ICU trial protocol.[19]

Primary statistical analysis plan: AID-ICU statistical analysis plan.[20]

Primary manuscript: Haloperidol for the Treatment of Delirium in ICU Patients.[21]

STROBE Statement—Checklist of items that should be included in reports of ***cohort studies***

	Item No	Recommendation
**Title and abstract**	1	(*a*) Indicate the study's design with a commonly used term in the title or the abstract – page 1
		(*b*) Provide in the abstract an informative and balanced summary of what was done and what was found – page 3
**Introduction**		
Background/rationale	2	Explain the scientific background and rationale for the investigation being reported – page 4
Objectives	3	State specific objectives, including any prespecified hypotheses – page 4
**Methods**		
Study design	4	Present key elements of study design early in the paper – page 5-7
Setting	5	Describe the setting, locations, and relevant dates, including periods of recruitment, exposure, follow-up, and data collection – page 5
Participants	6	(*a*) Give the eligibility criteria, and the sources and methods of selection of participants. Describe methods of follow-up – page 5 and ESM
		(*b*) For matched studies, give matching criteria and number of exposed and unexposed – not applicable
Variables	7	Clearly define all outcomes, exposures, predictors, potential confounders, and effect modifiers. Give diagnostic criteria, if applicable – page 5-6 and ESM
Data sources/ measurement	8*	For each variable of interest, give sources of data and details of methods of assessment (measurement). Describe comparability of assessment methods if there is more than one group – page 5, ESM and primary protocol and manuscript
Bias	9	Describe any efforts to address potential sources of bias – page 6-7
Study size	10	Explain how the study size was arrived at – primary statistical analysis plan [20]
Quantitative variables	11	Explain how quantitative variables were handled in the analyses. If applicable, describe which groupings were chosen and why – page 6-7, table 1 and ESM
Statistical methods	12	(*a*) Describe all statistical methods, including those used to control for confounding – page 6-7 and ESM
		(*b*) Describe any methods used to examine subgroups and interactions – page 6-7

		(*c*) Explain how missing data were addressed – page 7
		(*d*) If applicable, explain how loss to follow-up was addressed – pages 7
		(*e*) Describe any sensitivity analyses – not applicable
**Results**		
Participants	13*	(a) Report numbers of individuals at each stage of study—e.g. numbers potentially eligible, examined for eligibility, confirmed eligible, included in the study, completing follow-up, and analysed – page 8 and primary manuscript
		(b) Give reasons for non-participation at each stage – page 5, primary manuscript
		(c) Consider use of a flow diagram – primary manuscript
Descriptive data	14*	(a) Give characteristics of study participants (eg demographic, clinical, social) and information on exposures and potential confounders – Table 1 and primary manuscript
		(b) Indicate number of participants with missing data for each variable of interest – page 5
		(c) Summarise follow-up time (eg, average and total amount) – not applicable
Outcome data	15*	Report numbers of outcome events or summary measures over time – page 6-7, table 2, ESM
pMain results	16	(*a*) Give unadjusted estimates and, if applicable, confounder-adjusted estimates and their precision (eg, 95% confidence interval). Make clear which confounders were adjusted for and why they were included – table 2, page 6-7
		(*b*) Report category boundaries when continuous variables were categorized – page 6, primary protocol
		(*c*) If relevant, consider translating estimates of relative risk into absolute risk for a meaningful time period –table 2
Other analyses	17	Report other analyses done—eg analyses of subgroups and interactions, and sensitivity analyses – NA
**Discussion**		
Key results	18	Summarise key results with reference to study objectives – page 10
Limitations	19	Discuss limitations of the study, taking into account sources of potential bias or imprecision. Discuss both direction and magnitude of any potential bias – page 11-12
Interpretation	20	Give a cautious overall interpretation of results considering objectives, limitations, multiplicity of analyses, results from similar studies, and other relevant evidence – page 11-12
Generalisability	21	Discuss the generalisability (external validity) of the study results – primary manuscript
**Other information**		
Funding	22	Give the source of funding and the role of the funders for the present study and, if applicable, for the original study on which the present article is based – page 1

*Give information separately for exposed and unexposed groups.

Note: An Explanation and Elaboration article discusses each checklist item and gives methodological background and published examples of transparent reporting. The STROBE checklist is best used in conjunction with this article (freely available on the Web sites of PLoS Medicine at http://www.plosmedicine.org/, Annals of Internal Medicine at http://www.annals.org/, and Epidemiology at http://www.epidem.com/). Information on the STROBE Initiative is available at http://www.strobe-statement.org

References

1. Ely EW, Margolin R, Francis J, et al. Evaluation of delirium in critically ill patients: validation of the Confusion Assessment Method for the Intensive Care Unit (CAM-ICU). Critical care medicine 2001;29(7):1370-9. (http://www.ncbi.nlm.nih.gov/pubmed/11445689).
2. Bergeron N, Dubois MJ, Dumont M, Dial S, Skrobik Y. Intensive Care Delirium Screening Checklist: evaluation of a new screening tool. Intensive care medicine 2001;27(5):859-64. (http://www.ncbi.nlm.nih.gov/pubmed/11430542).
3. Sessler CN, Gosnell MS, Grap MJ, et al. The Richmond Agitation-Sedation Scale: validity and reliability in adult intensive care unit patients. American journal of respiratory and critical care medicine 2002;166(10):1338-44. DOI: 10.1164/rccm.2107138.
4. Ramsay MA, Savege TM, Simpson BR, Goodwin R. Controlled sedation with alphaxalone-alphadolone. Br Med J 1974;2(5920):656-9. DOI: 10.1136/bmj.2.5920.656.
5. Devlin JW, Boleski G, Mlynarek M, et al. Motor Activity Assessment Scale: a valid and reliable sedation scale for use with mechanically ventilated patients in an adult surgical intensive care unit. Critical care medicine 1999;27(7):1271-5. DOI: 10.1097/00003246-199907000-00008.
6. Teasdale G, Jennett B. Assessment of coma and impaired consciousness. A practical scale. Lancet 1974;2(7872):81-4. DOI: 10.1016/s0140-6736(74)91639-0.
7. Riker RR, Picard JT, Fraser GL. Prospective evaluation of the Sedation-Agitation Scale for adult critically ill patients. Critical care medicine 1999;27(7):1325-9. DOI: 10.1097/00003246-199907000-00022.
8. Granholm A, Kaas-Hansen BS, Lange T, et al. Use of days alive without life support and similar count outcomes in randomised clinical trials - an overview and comparison of methodological choices and analysis methods. BMC Med Res Methodol 2023;23(1):139. DOI: 10.1186/s12874-023-01963-z.
9. Andersen-Ranberg N. Effect of haloperidol on hypoactive and hyperactive delirium – a secondary post-hoc study of the AID-ICU trial -statisical outline. OSF Registries. (https://osf.io/q6mdt/).
10. Bürkner P-C. brms: An R package for Bayesian multilevel models using Stan. Journal of statistical software 2017;80(1):1-28.
11. Andersen-Ranberg NC, Poulsen LM, Perner A, et al. Haloperidol vs. placebo for the treatment of delirium in ICU patients: a pre-planned, secondary Bayesian analysis of the AID–ICU trial. Intensive care medicine 2023. DOI: 10.1007/s00134-023-07024-9.
12. Carpenter B, Gelman A, Hoffman MD, et al. Stan: A probabilistic programming language. Journal of statistical software 2017;76(1):1-32.
13. Gelman A CJ, Stern HS, Dunson DB, Vehtari A, Rubin DB. Bayesian Data Analysis, 3rd edn. London, UK: Chapman and Hall/ CRC, 2013.
14. Vehtari A GA, Simpson D, Carpenter B, Bürkner P- C. Rank-normalization, folding, and localization: An improved Rhat for as- sessing convergence of MCMC. arXiv 2020:190308008v3 [stat CO] 2020.
15. Gabry J, Simpson D, Vehtari A, Betancourt M, Gelman A. Visualization in Bayesian workflow. arXiv preprint arXiv:170901449 2017.
16. Vehtari A GA, Gabry J. Practical Bayesian model evaluation using leave- one- out cross-validation and WAIC. Stat Comput 2017;27:1413-1432.

17. Vehtari A. Cross-validation FAQ. ([https://avehtari.github.io/modelselection/CV-FAQ.html]).
18. Granholm A, Munch MW, Myatra SN, et al. Dexamethasone 12 mg versus 6 mg for patients with COVID-19 and severe hypoxaemia: a pre-planned, secondary Bayesian analysis of the COVID STEROID 2 trial. Intensive care medicine 2022;48(1):45-55. DOI: 10.1007/s00134-021-06573-1.
19. Andersen-Ranberg NC, Poulsen LM, Perner A, et al. Agents intervening against delirium in the intensive care unit (AID-ICU) - Protocol for a randomised placebo-controlled trial of haloperidol in patients with delirium in the ICU. Acta anaesthesiologica Scandinavica 2019;63(10):1426-1433. (In eng). DOI: 10.1111/aas.13453.
20. Andersen-Ranberg N, Poulsen LM, Perner A, et al. The Agents Intervening against Delirium in the Intensive Care Unit Trial (AID-ICU trial): A detailed statistical analysis plan. Acta anaesthesiologica Scandinavica 2020;64(9):1357-1364. DOI: 10.1111/aas.13661.
21. Andersen-Ranberg NC, Poulsen LM, Perner A, et al. Haloperidol for the Treatment of Delirium in ICU Patients. The New England journal of medicine 2022;387(26):2425-2435. DOI: 10.1056/NEJMoa2211868.
